# Automating document classification for the Immune Epitope Database

**DOI:** 10.1186/1471-2105-8-269

**Published:** 2007-07-26

**Authors:** Peng Wang, Alexander A Morgan, Qing Zhang, Alessandro Sette, Bjoern Peters

**Affiliations:** 1 The La Jolla Institute for Allergy and Immunology, 9420 Athena Circle, La Jolla, CA 92037, USA; 2 Biomedical Informatics, Stanford University School of Medicine, Stanford, California 94305-5120, USA

## Abstract

**Background:**

The Immune Epitope Database contains information on immune epitopes curated manually from the scientific literature. Like similar projects in other knowledge domains, significant effort is spent on identifying which articles are relevant for this purpose.

**Results:**

We here report our experience in automating this process using Naïve Bayes classifiers trained on 20,910 abstracts classified by domain experts. Improvements on the basic classifier performance were made by a) utilizing information stored in PubMed beyond the abstract itself b) applying standard feature selection criteria and c) extracting domain specific feature patterns that e.g. identify peptides sequences. We have implemented the classifier into the curation process determining if abstracts are clearly relevant, clearly irrelevant, or if no certain classification can be made, in which case the abstracts are manually classified. Testing this classification scheme on an independent dataset, we achieve 95% sensitivity and specificity in the 51.1% of abstracts that were automatically classified.

**Conclusion:**

By implementing text classification, we have sped up the reference selection process without sacrificing sensitivity or specificity of the human expert classification. This study provides both practical recommendations for users of text classification tools, as well as a large dataset which can serve as a benchmark for tool developers.

## Background

Manual curation of information from the literature into electronic databases is of increasing importance in biomedical science. Prominent examples of databases incorporating curated data include the Swiss-Prot section of the Universal Protein Resource Knowledgebase (UniProtKB/Swiss-Prot) [1], the Gene Reference Into Function (GeneRIF) system [2], the Mouse Genome Informatics Database [3], KEGG [4], DIP [5] and BIND [6]. These databases provide targeted query interfaces to access their data, which allow the performance of summary analysis that would be much harder or impossible to perform when relying on literature alone.

This study was motivated by the needs of the Immune Epitope Database and Analysis Resource (IEDB) [7,8]. The IEDB catalogs molecular structures of immune epitopes recognized by the antigen specific receptors of T cells and B cells or binding to the major histocompatibility complex (MHC) molecules. In addition, the IEDB describes the biological context in which epitope recognition was recorded including information on the host organism, immunogen, antigen and assay. Together with the analytical tools hosted within it, the IEDB provides resources for the development of epitope based techniques to detect, monitor, and prevent or treat diseases.

The IEDB literature curation is a highly iterative and cooperative process [9]. Epitope-related references are first extracted from PubMed through complex queries consisting of multiple keywords and logical operators. Since the queries were designed to return all potentially relevant references, a substantial number of abstracts returned by the query are not actually in the scope of the IEDB and need to be removed from the curation process. Examples for studies that are out of the IEDB scope, even though their abstracts contain epitope-related keywords, include those describing a) epitope-tags for protein purification purposes, b) computational studies describing epitope predictions but not experiments, and c) studies which characterize immune responses without identifying the molecular structure of the targeted epitopes. Until recently, this abstract classification was performed manually by two experienced immunologists. Abstracts deemed to be outside of the IEDB scope are discarded. For abstracts within the scope, the full-text articles are retrieved and their epitope related content is entered into the database by a team of curators with expertise in biochemistry, microbiology and immunology. The accuracy of the curation is reviewed by an independent panel of senior immunologists and structural biologists, which give final approval to make the data public on the IEDB website.

Manual literature curation is a resource and time-consuming process, which makes it highly desirable to automate any part of it. One step that clearly lends itself for automation is determining which publication retrieved from PubMed is likely to contain relevant information. This is referred to as text classification or relevance assessment and falls in the scientific domain of information retrieval [10,11]. The most prominent platform to evaluate the performance of information retrieval techniques is the Text Retrieval Conference (TREC) organized by the US national Institute for Standards and Technology (NIST). With the recent addition of TREC's genomics track [12], TREC serves as a valuable resource to promote the advance and application of information retrieval techniques towards real world biomedical problems.

Text classification is one of the simplest information retrieval procedures [11] and can be directly applied to the IEDB reference selection process. Early attempts of text classification were mainly carried out by constructing manually designed rules engineered from expert knowledge [13]. With the increased availability of large amount of literature in digital format, machine learning methods became the dominant approaches. A large body of literature has been published documenting the application of virtually every major machine learning algorithms in text classification [11,14]. Popular approaches include Naïve Bayes Classifiers [15], Decision Trees [16] and Support Vector Machines (SVM) [17].

Automated text classification has been successfully applied to aid in biomedical database curation. Donaldson et al. [18] used an SVM model to distinguish abstracts containing information on protein-protein interaction before they were curated into the BIND database. In related work, a Probabilistic Latent Categoriser (PLC) with Kullback-Leibler (KL) divergence was used to re-rank PubMed references before they were curated into the SWISS-PROT database [19]. Miotto et al. used artifical neural networks and classification trees to identify relevant abstracts containing allergen cross-reactivity information [20]. In a more recent study, Chen et al. combined SVM with a novel phrase-based clustering algorithm to classify papers about *C. elegans *[21].

We here report the implementation of automated text classification via a Naïve Bayes Classifier into the IEDB curation process. The Naïve Bayes Classifier approach is a popular machine learning method for text classification because it is fast, easy to implement and performs well. It has a long and distinguished record in text classification [22,23] and it has been successfully applied to real world problems such as filtering spam emails [24]. The classification algorithms were developed utilizing the large dataset of 20,910 PubMed abstracts previously classified by experts. Several established text classification techniques were compared, and a Naïve Bayes classifier with information theory based feature selection performed best. This classifier was further customized with domain-specific approaches to feature selection. The final algorithm was put into practice classifying abstracts as "relevant", "irrelevant" and "uncertain", of which only the latter are further reviewed by an expert. This greatly reduces the workload for the human expert without sacrificing classification performance.

## Results

### Overview of the dataset

The dataset used in our study are 20,910 PubMed abstracts that have been manually evaluated by domain experts during the population of the IEDB in the period from October 2005 to October 2006 (Table 1). The IEDB is consecutively targeting different categories of epitope information based on different epitope sources: category A-C pathogens (AC), emerging and re-emerging diseases (ER), allergens, and epitope sources not in the previous three categories (other). The abstracts were extracted from PubMed using complex keyword queries tailored for each category [see Additional file 1].

**Table 1:** Summary of expert curated abstracts used in this study. The abstracts are partitioned into four categories based on the pathogen from which the epitopes are derived. Other refers to the pathogens that are not in the first three categories

**Category**	**Number of abstracts**	**Relevant abstracts**	**Irrelevant abstracts**
**Category A-C pathogens**	7577	1942	**5635**
**Allergen**	4992	1080	**3912**
**Emerging and Re-emerging infectious diseases**	2911	845	**2066**
Other	**5430**	**1845**	**3585**

All abstracts are expert classified as either "relevant" or "irrelevant" to the scope of the IEDB [see Additional file 2]. "Relevant" abstracts are abstracts that contain epitope related information that can be extracted and curated into the IEDB. The majority of abstracts returned in the PubMed query are not relevant. The relevant:irrelevant ratios range from 1:2 for AC pathogens to 1:4 for allergens and there are a total of 5,346 relevant and 15,564 irrelevant references. Of course, the human expert classifications are not always correct. This is especially true as the abstract scanning is done very rapidly. To estimate the inherent disagreement rate in this process, we compared the classifications made by two experts on the same dataset. We found the rate of disagreement to be slightly less than 5%. This inherent disagreement rate in our dataset limits the accuracy that can be achieved even from a perfect classification algorithm.

### Application of established text classification techniques

**Figure 1:**
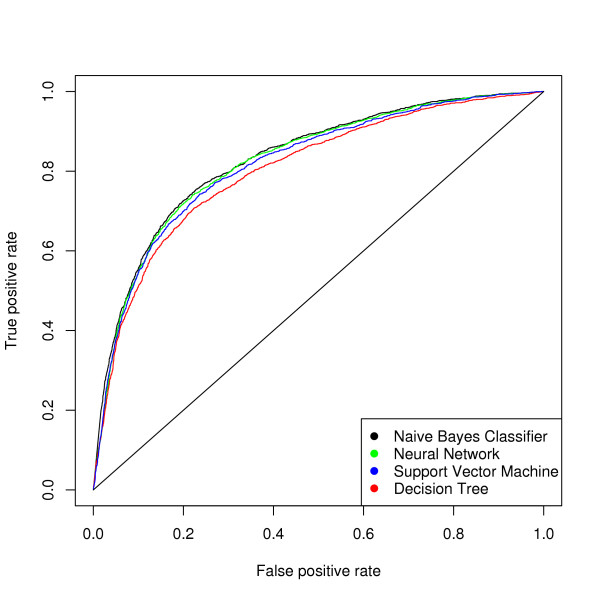
**Comparison of popular text classification algorithms**. The performance of four well-known text classification algorithm was evaluated on our dataset via 10-fold cross-validation. The ROC curve shows that the Naïve Bayes classifier performs best on our dataset. The AUC values for the four classifiers are as follows: Naïve Bayes classifier: 0.838; Neural Network: 0.831; Support Vector Machine: 0.825; Decision Tree: 0.809.

We first evaluated the performance of a number of standard text classification algorithms using their default implementation in the WEKA package [25]. Their performance was compared in 10-fold cross-validation (Figure 1) using area under receiver operating characteristic curves (AUC) as the performance metric. The input features for classification are the raw words in each abstract. We adopted a set of words approach and treat the features as binary attributes. In other words, we take into account only the presence of a feature in an abstract, not the number of times it occurs. All features were used except for "stop words", common words which occurred at identical frequency in relevant and irrelevant abstracts (e.g. "and", "with", "a", "the"). Of the tested algorithms, the Naïve Bayes classifier performed best (AUC = 0.838) for our dataset. To increase our flexibility in modifying the algorithm and to avoid computer memory issues with the WEKA package in handling very large datasets, we implemented the classification and performance evaluation as a set of python scripts. The classification performance was identical with that of the WEKA package. The implemented classifier also has fast executing speed and can finish 10-fold cross-validation on the 20,910 abstracts in about 15 minutes when tested on Ubuntu Linux system with a 2 GHz Pentium 4 processor. When the trained classifier is used to process new abstracts, it can classify 1,000 abstracts in less than 30 seconds.

**Figure 2:**
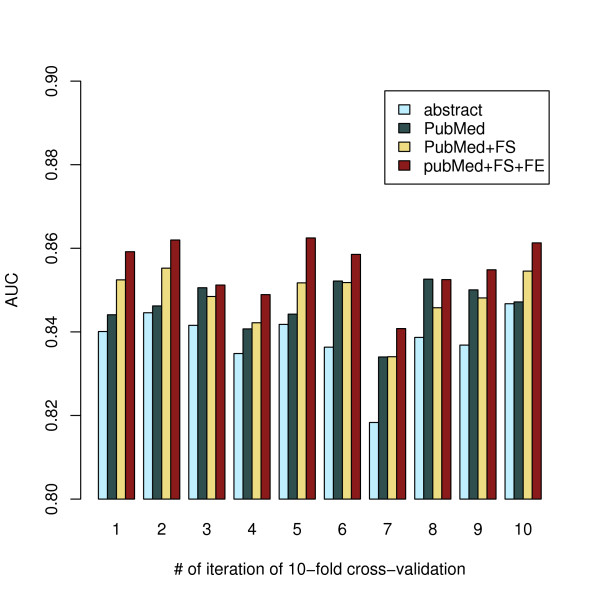
**Comparison of Naive Bayes Classifier performance in cross validation**. AUCs of Naïve Bayes classifier incorporating various dimensionality reduction techniques were compared in each round of the 10-fold cross-validation side by side. **Abstract: **AUC of classifier trained on the raw words of abstracts. **PubMed: **AUC of classifier trained on raw words in abstract, MeSH heading, title, author etc. **PubMed+FS: **AUC of classifier trained on subset of raw words selected from abstract, MESH heading, title, author etc using combined cutoff of IG >2.00e-05 and DF >3. **PubMed+FS+FE: **AUC of classifier trained on a subset of feature generated from raw words in abstract, MeSH heading, title, author etc by first applying feature extraction followed by feature selection. Using combined cutoff of IG >2.00e-05 and DF >3.

The features that can be extracted from PubMed include not only the words in the abstract and title, but also the authors, the journal and MeSH headings. We compared using all of these features with using only the words of the abstract. The ten-fold cross-validation estimated performance improved from 0.838 to 0.846 (Figure 2) which is significant with a p-value 0.0021 as determined by a paired t-test (see Methods). This improvement in classifier performance clearly demonstrates that the abstract contains most but not all of the information useful for reference classification.

While the t-test shows that the two methods produce different AUC values, one can reasonably ask if a change of 0.008 in AUC has any practical relevance. We can test the improvement by using the irrelevant abstracts in our dataset. In practice we wish to remove as many as possible the irrelevant abstracts while retaining 95% of the relevant abstracts. The classifier with AUC of 0.838 applied to our dataset of 20,910 abstracts will identify 9,716 false positive abstracts to achieve a 95% true positive rate, while the classifier with AUC of 0.846 will identify 9,421 false positive abstracts. This results in a net decrease of 295 false positive abstracts and is a performance improvement of 3%, which is a small but noticeable practical improvement.

**Figure 3:**
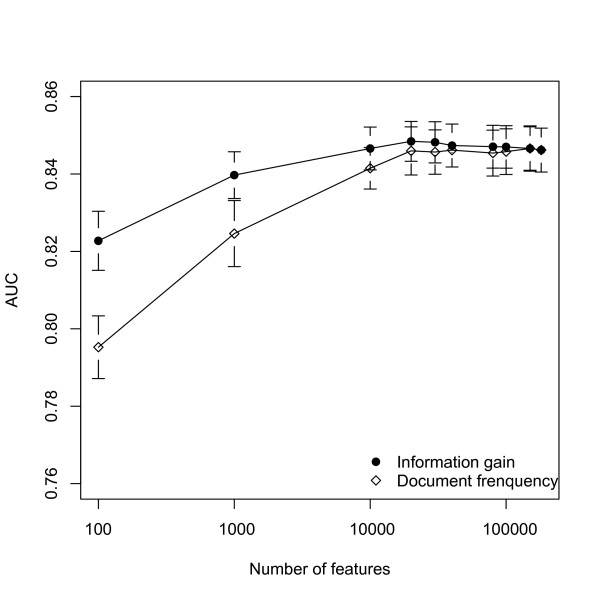
**Effects of feature selection on Naïve Bayes classifier performance**. The performances of the Naïve Bayes classifier (measured in AUC) is plotted against the number of features used in training. Both IG (information gain) and DF (document frequency) based feature selection have a similar effect on classifier performance. Reducing the number of features used to the top 20,000 by each measure leads to a small increase in performance. Using even less features leads to decreases in performance, but notably the top 100 features in term of information gain are sufficient to reach AUC values of 0.82.

The feature space in our dataset is now consisting of 181,299 unique words. To select features that are likely to be more relevant, we applied two well accepted feature selection methods: document frequency (DF) and information gain (IG) described in the **Methods **section. The performance curves of Naïve Bayes classifier after feature selection were plotted in Figure 3. Both IG and DF feature selection methods have similar effects on the classifier performance. The best performances are achieved when around 20,000 features are used. Both techniques permit removing up to 80% of the features while maintaining improved performance under cross validation. Applying the combined cutoff with DF>3 and IG>2.00e-05 selects 20,509 features and increases the AUC in ten-fold cross-validation from 0.846 to 0.848 (p = 0.22) (Figure 2). While this improvement is not significant when applying the customary p < 0.05 cutoff, it is a benefit in itself to remove features carrying little information or occurring rarely, leading to decreases in computation time and a reduction in the risk of over fitting.

An alternative approach to condense the feature space is to identify words by their stem, e.g. reducing the words "binding, binds, bind" to their common stem "bind". There are standard stemming algorithms designed to handle everyday English writing [26], which are often applied for text classification including for bioinformatics. When we applied the Porter stemming algorithm instead of feature selection, we found that the classifier performance actually drops significantly from AUC = 0.846 to AUC = 0.842 (p = 0.036) (Figure 2). This was in agreement with a previous study [27] that suggests the standard Porter stemmer may not be suitable to text classification for biomedical literature, as there is a large set of domain specific vocabulary which may be reduced to unsuitable stems. We did not test alternative stemming algorithms, some of which are listed in the discussion section.

### Domain specific feature extraction

In order to reduce the dimensionality of the feature space while still capturing the essence of domain specific features, we introduced novel rules that specifically try to capture immune epitope related expressions, and group them together. Through this process, the information carried in individual expressions is combined and the information content is enriched which can lead to better performance. The following four concepts were identified:

(1) Peptide sequences (e.g. "SIINFEKL" or "ALTFVWGMKR"). Sequences of peptides are often included in abstracts describing epitopes, but make poor features for text classification as two sequences that are not identical letter by letter will be treated as separate features. We identified peptide sequences as a) exclusively containing characters representing the twenty amino acids, b) in upper case c) length greater than seven and d) not one of the following words: "CLINICAL", "MATERIAL", "MATERIALS", "PATIENTS", "RESEARCH" or "SIGNIFICANCE". All identified peptide sequences are replaced with "~peptide~ ".

(2) Position ranges (e.g. "276–284"). Expressions that can indicate the location of an epitope in a protein sequence were identified and replaced with "~range<50~ " or "~range>50~ " depending on the length of the range specified. As the IEDB requires epitopes to be mapped to stretches of less than 50 amino acids, only ranges of less than 50 are good indicators of relevance.

(3) MHC alleles (e.g. "HLA A*0201" or "H-2 Db") There are thousands of different MHC alleles described with different nomenclatures and numbering systems for different species. We compiled a regular expression representing most MHC alleles from humans and mice and replaced them with "~mhc_allele~ ".

(4) "X-mers" (e.g. "9-mer", "15-mer), which is a term referring to peptides of a specific length. The X-mers were identified with regular expressions matching the following pattern: one or more digits followed by hyphen then followed by "mer", and were translated into "~#integer~-mer".

The addition of domain specific feature extraction consistently improved the performance of the classifier (Figure 2). The Naïve Bayes classifier using all features from PubMed (abstracts, MeSH heading, titles etc) and feature selection has an average AUC of 0.848. Incorporating domain specific feature extraction further improves its performance to an AUC of 0.855, which is highly significant (p = 1.3E-06).

Table 2 lists the top features selected by information gain. Several features identified via our feature extraction rules turned out to be highly informative. Extracted concepts like ~peptide~, ~mhc-allele~ are on par with the most informative words utilized by domain experts such as "epitope". Features that are strong indication of "irrelevant" are also informative. "Superantigens", which induce immune response without involvement of epitopes, ranked as the 275^th ^among all features with an information gain of 0.0012. "Epitope-tagged", which usually indicates protein engineering experiments, ranks as the 106^th ^among all features with an information gain of 0.00236. The improvements from utilizing additional features from PubMed are also reflected in the top ranking features. Table 3 lists top MeSH headings selected by information gain. These MeSH headings are highly informative as judged by information gain, and ranked as high as 21st among all features. An interesting observation is that author names can also be valuable features. For example the name "Rammensee" ranked 393rd among all features with an information gain of 0.000845.

**Table 2:** Top features selected via information gain. Column one is the feature. Column two is the average IG (information gain) of the feature calculated from 10-fold cross-validation. Column 3 is the feature's DF (document frequency) calculated from the whole dataset. Only features with an IG greater that 0.01 are shown

**Feature**	**IG**	**DF**
Epitope	0.0441	5707
Peptide	0.0402	6408
Amino	0.0369	6461
Sequence	0.0308	6849
Acid	0.0289	6633
~range<50~	0.0247	2915
Synthetic	0.0242	1878
~mhc_allele~	0.0228	2745
overlapping	0.0174	781
Recognized	0.0159	3097
immunodominant	0.0153	1483
Mapping	0.0146	1433
Residues	0.0144	2108
Molecular	0.0126	7405
~peptide~	0.0118	610

**Table 3:** Top 10 MeSH heading features selected via information gain. MeSH terms with asterisk * denote major MeSH terms referring to the "major" topic that is discussed in the article as defined by the MeSH curator

**Feature**	**IG**	**DF**
epitopes/*immunology	0.00664	682
cytotoxic/*immunology	0.00604	746
peptides/chemical	0.00462	262
synthesis/immunology	0.00394	203
t-lymphocytes/*immunology	0.00388	1236
Hepacivirus/*immunology	0.00361	188
fragments/*immunology	0.00311	280
Proteins/*immunology	0.00269	958
peptides/immunology	0.00253	317
Antigens/*immunology	0.00244	501

### Classification of abstracts from a new sub-domain

As described above, the IEDB consecutively curates abstracts from different sub-domains based on the source of the epitope. This means that the cross-validation used here could overestimate the performance of the classifier whenever abstracts from a new sub-domain are being classified. To test the performance of existing classifiers on newly acquired abstracts from a different sub-domain, we performed a series of tests. In each of such test, we first trained a classifier based on three of the four available sets of abstracts. We then tested the performance of classifiers learn on such sets on the fourth set of abstracts (Table 4). The tests demonstrated that Naïve Bayes classifiers learned from different categories of abstracts have significantly lower but still competitive performances and can achieve AUCs in the range of 0.784 to 0.852. These lower performance estimates have to be used when applying the classifier to a new sub-domain of articles, and were used to establish the cutoff values in the following section.

**Table 4:** Classifier performances on sub-domains. The entries of the table are AUC values of Naïve Bayes classifier performance results. The AUC was derived by training the Naïve Bayes classifier on a dataset (row) and use the model learned to classify a second dataset (column). The first row of numbers is the AUC from 10-fold cross-validation. The second row of numbers is the AUC values when the classifiers are trained using abstracts from other categories

	**Classification performance on sub-domain (AUC)**			
**Train Set**	**AC**	**Allergen**	**ER**	**Other**

**Sub-domain (cross-validation)**	0.847	0.856	0.858	0.861
**All other sub-domains**	0.797	0.817	0.852	0.784

### Testing text classification in practice: the malaria abstracts

Our goal for the classification is to remove as many irrelevant abstracts as possible while maintaining a false negative rate comparable to the human expert disagreement rate of 5%. We also do not want to overload the curation queue with false positive abstracts, as placing articles in the queue and later removing them involves costs for e.g. retrieving the full text manuscripts. We therefore classify references in one of three categories: Abstracts with very high predictive scores (cutoff: +100) predominantly relevant to the IEDB and are directly placed into the curation pipeline. Abstracts with very low predictive scores (cutoff: -100) are predominantly irrelevant to the IEDB and can be safely discarded. Those abstracts with intermediate predictive scores are manually classified by domain experts. The thresholds determining what scores are very high and very low are chosen based on the performance in classifying abstracts from a new sub-domain described above.

**Figure 4:**
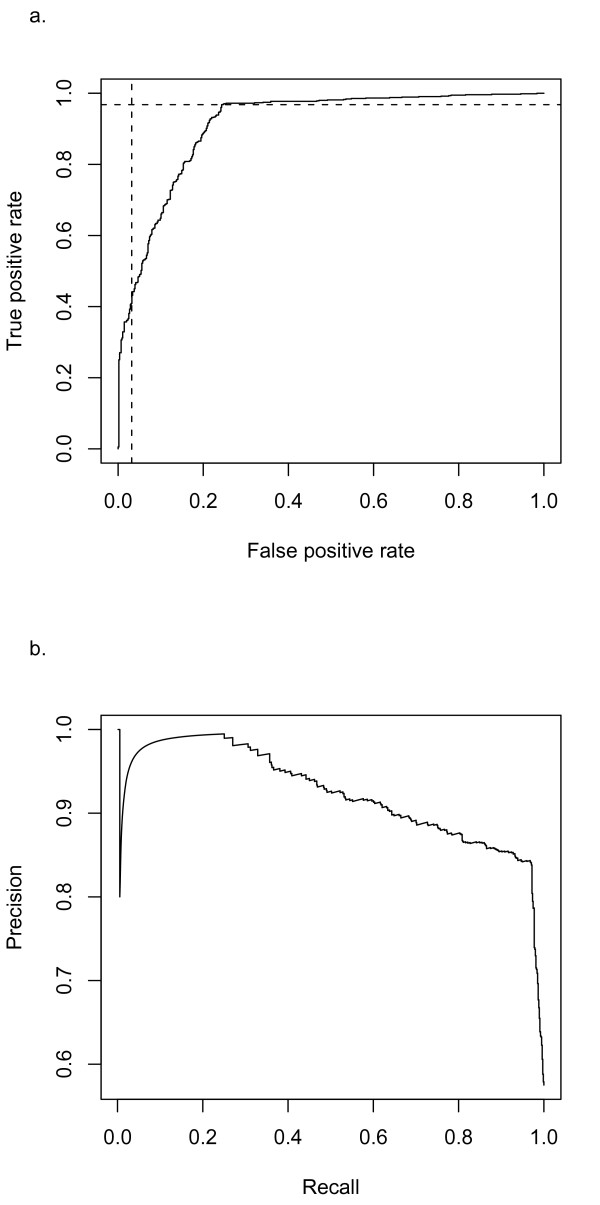
**ROC and Precision-recall curve of malaria abstracts**. Newly acquired malaria abstracts were classified with the Naïve Bayes classifier trained on all previously expert classified abstracts. The ROC curve was shown in Figure 4a. Horizontal line is the cutoff for "irrelevant" abstracts and vertical line is the cutoff for "relevant" abstracts. Figure 4b is the Precision-Recall curve. The curve shows that at 95% precision, we achieved a recall rate of 36.4%.

The first test case of this scheme was the classification of abstracts from malaria epitopes, which constitutes a new sub-domain. The initial PubMed query returned 1,470 abstracts with malaria epitope specific keywords. We classified the malaria abstracts using the Naïve Bayes classifier trained on all available abstracts and validated the predicted against manual classification results. A ROC plot and precision-recall graph of the classification performance is shown in Figure 4. There are 310 abstracts classified as "relevant" according to the cutoff determined from previous training. A close examination shows that 295 out of those 310 abstracts are also classified as "relevant" by domain experts (Table 5) which gives a positive prediction value of 95.2%. We next examined the 441 abstracts have been classified into the irrelevant category using cutoff determined from previous training (Table 4). A comparison shows that the negative predictive value for this cutoff is 94.8% which is right on par with domain experts. In summary, using classifiers built on existing abstracts, we were able to classify 51.1% of newly acquired malaria abstracts with performance on par with human experts (Table 5).

**Table 5:** Classifier performance on newly acquired malaria abstracts

	Relevant abstracts predicted by classifier (310)	Irrelevant abstracts predicted by classifier (441)
Relevant abstracts determined by Domain expert	295	23
Irrelevant abstracts determined by Domain expert	15	418

A close examination of miss-classified abstracts can reveal interesting insights. For example, the abstract with PMID 16791622 was classified as relevant with a high score of 162.3 while it is in fact irrelevant to the IEDB. The authors discuss the sequence and structure of MHC molecules of Aotus monkeys and compare their peptide binding region with that of human MHC molecules. However, no experimental data was generated. This example shows the limitation of the independency assumption of the Naïve Bayes classifier and suggests that more sophisticated methods are required to classify such abstracts automatically.

## Discussion

We report here our implementation of text classification into the IEDB curation process. Using cross-validation on existing data to evaluate classifier performances, we built a customized Naïve Bayes classifier to categorize if PubMed abstracts are within the scope of the IEDB. The final classification scheme was applied to a set of abstracts from a new sub-domain of epitopes, and successfully validated.

A number of lessons were learned that are generally applicable for similar projects and may not be immediately obvious. First of all, we found classification performance was improved when using information beyond abstract and title of a study such as MeSH terms and the names of authors conducting a study. Author names are not commonly included in text classification, but doing so makes sense as some scientists specialize in the application of methods, which makes their authorship of a paper indicative of its content. Potentially, there are many more sources of information linked to a manuscript which could be used as additional features for classifications. For example, if an article is the primary reference for an entry in the PDB, the information contained in the PDB could be used as a source for features. Similarly, the references citing or cited by an article could be incorporated into the feature space.

Secondly, we found that grouping together biomedical terms with the same meaning has a significant positive impact on classifier performance. Grouping not only reduced the dimensionality of the feature space but also created features with enriched information that contributed to better performance. Several of the features we extracted are likely to be relevant for other domains as well, e.g. a project doing transplant research could benefit from our regular expressions used for identifying MHC alleles.

Finally, we have implemented a hybrid categorization process using the automated classifier to pre-group abstracts into clearly relevant, clearly irrelevant, or uncertain. The latter ones are then classified by a human expert. This stream of expert classifications will be used to continuously update the classifier, which should result in improvements in performance that will further reduce the number of abstracts for which human classification is necessary.

There are a number of ways the classifier could be further improved. For example, we only tested one word stemming algorithm, the Porter stemmer. The use of less stringent stemming algorithms (such as Krovetz stemmer [28]) or a combination of stemming and part of speech tagging could help to improve classifier performance as this would tend to preserved domain specific terms and has been useful in information retrieval from PubMed [29]. We could also incorporate techniques learned from information retrieval research in other domains [11]. For example, potential improvements may be achieved by giving higher weight to documents having passages with high concentrations of high information content terms. Also, the high information content terms identified in this study could be applied to refine the construction of the initial PubMed queries. Many irrelevant abstracts are returned by our current queries; this could potentially be avoided if more discriminating search terms can be identified.

In addition to reporting what we believe to be valuable lessons learned, we also make the accompanying datasets of expert classified abstracts publicly available (Supplemental Material 2), which could be a valuable addition for existing resources benchmarking biomedical text classification. The BioCreAtIvE (Critical Assessment of Information Extraction systems in Biology) provides several such benchmarks for information extraction and text mining [30]. The PubMed abstracts annotated with MeSH terms are another resource of expert classified abstracts. Comparing to benchmarks that are currently available, our abstracts offer a very large dataset for possible optimal training of classifiers to address a practical use-case. It is critical for the development and evaluation of biomedical text mining and categorization tools to identify biologically significant problems and set up corresponding benchmarks for evaluation. This will benefit the community just like what the CASP evaluation has contributed to computational protein structure. We want to strongly encourage others to utilize the IEDB dataset as part of their benchmarking, and hope to learn from their experience to further improve our process.

## Conclusion

In summary, we have successfully sped up the abstract selection process of IEDB reference curation. We achieved sensitivity and specificity comparable to that of the human expert classification on a subset of automatically classified abstracts by combining standard machine learning techniques and novel feature extraction method and using a hybrid machine/human classification scheme. The insights learned from this study provide practical recommendations for users of text classification tools and the large dataset can serve as a benchmark to facilitate progress in tool development.

## Methods

### Feature selection

Two filter type feature selection algorithms are utilized. Document frequency (DF) is a simple and efficient methods where features are ranked based on the number of abstracts they appear in. Information gain (IG) is an information theory based approach. It measures the number of bits of information obtained for category prediction by knowing the presence or absence of a feature in a document. The definition is listed below,*t *denotes the feature of interest, *c*_*i *_(*i *= 1,..., *m*) denotes the set of categories the documents belong to. Each feature in the training set is evaluated in terms of DF and IG, and features falling below the cutoff are excluded from the training process.

IG(t)=−∑i=1mP(ci) log P(ci)+P(t)∑i=1mP(ci|t) log P(ci|t)+P(t¯)∑i=1mP(ci|t¯) log P(ci|t¯)
 MathType@MTEF@5@5@+=feaafiart1ev1aaatCvAUfKttLearuWrP9MDH5MBPbIqV92AaeXatLxBI9gBaebbnrfifHhDYfgasaacH8akY=wiFfYdH8Gipec8Eeeu0xXdbba9frFj0=OqFfea0dXdd9vqai=hGuQ8kuc9pgc9s8qqaq=dirpe0xb9q8qiLsFr0=vr0=vr0dc8meaabaqaciaacaGaaeqabaqabeGadaaakeaacqWGjbqscqWGhbWrdaqadaqaaiabdsha0bGaayjkaiaawMcaaiabg2da9iabgkHiTmaaqahabaGaemiuaa1aaeWaaeaacqWGJbWydaWgaaWcbaGaemyAaKgabeaaaOGaayjkaiaawMcaaaWcbaGaemyAaKMaeyypa0JaeGymaedabaGaemyBa0ganiabggHiLdGccqqGGaaiieGacqWFSbaBcqWFVbWBcqWFNbWzcqqGGaaicqWGqbaudaqadaqaaiabdogaJnaaBaaaleaacqWGPbqAaeqaaaGccaGLOaGaayzkaaGaey4kaSIaemiuaa1aaeWaaeaacqWG0baDaiaawIcacaGLPaaadaaeWbqaaiabdcfaqnaabmaabaGaem4yam2aaSbaaSqaaiabdMgaPbqabaGccqGG8baFcqWG0baDaiaawIcacaGLPaaaaSqaaiabdMgaPjabg2da9iabigdaXaqaaiabd2gaTbqdcqGHris5aOGaeeiiaaIae8hBaWMae83Ba8Mae83zaCMaeeiiaaIaemiuaa1aaeWaaeaacqWGJbWydaWgaaWcbaGaemyAaKgabeaakiabcYha8jabdsha0bGaayjkaiaawMcaaiabgUcaRiabdcfaqnaabmaabaGafmiDaqNbaebaaiaawIcacaGLPaaadaaeWbqaaiabdcfaqnaabmaabaGaem4yam2aaSbaaSqaaiabdMgaPbqabaGccqGG8baFcuWG0baDgaqeaaGaayjkaiaawMcaaaWcbaGaemyAaKMaeyypa0JaeGymaedabaGaemyBa0ganiabggHiLdGccqqGGaaicqWFSbaBcqWFVbWBcqWFNbWzcqqGGaaicqWGqbaudaqadaqaaiabdogaJnaaBaaaleaacqWGPbqAaeqaaOGaeiiFaWNafmiDaqNbaebaaiaawIcacaGLPaaaaaa@913B@

### Naïve Bayes Classifier

The Bayesian approach for classification is to assign a new instance *v*_*j *_the most probable target value *v*_*target *_given a set of attribute values <α_1_, α_2_... α_*n*_> describing the instance and a set of previously classified instances with known attributes. The Naïve Bayes classifier is based on the simplifying assumption that the attribute values are conditionally independent given the target value [31]. Based on this assumption, we can assign the target class as following:

Vtarget=argmaxvj∈VP(vj)∏i=1nP(ai|vj)
 MathType@MTEF@5@5@+=feaafiart1ev1aaatCvAUfKttLearuWrP9MDH5MBPbIqV92AaeXatLxBI9gBaebbnrfifHhDYfgasaacH8akY=wiFfYdH8Gipec8Eeeu0xXdbba9frFj0=OqFfea0dXdd9vqai=hGuQ8kuc9pgc9s8qqaq=dirpe0xb9q8qiLsFr0=vr0=vr0dc8meaabaqaciaacaGaaeqabaqabeGadaaakeaacqWGwbGvdaWgaaWcbaGaemiDaqhcbiGae8xyaeMae8NCaiNae83zaCMaemyzauMaemiDaqhabeaakiabg2da9maaxababaGae8xyaeMae8NCaiNae83zaCMae8xBa0Mae8xyaeMae8hEaGhaleaacqWG2bGDdaWgaaadbaGaemOAaOgabeaaliabgIGiolabdAfawbqabaGccqWGqbaudaqadaqaaiabdAha2naaBaaaleaacqWGQbGAaeqaaaGccaGLOaGaayzkaaWaaebCaeaacqWGqbaudaqadaqaaiabdggaHnaaBaaaleaacqWGPbqAaeqaaOGaeiiFaWNaemODay3aaSbaaSqaaiabdQgaQbqabaaakiaawIcacaGLPaaaaSqaaiabdMgaPjabg2da9iabigdaXaqaaiabd6gaUbqdcqGHpis1aaaa@5C3B@

We implemented the multinomial model version of Naïve Bayes classifier with python scripts for maximum flexibility. In addition, the WEKA [25] implementation of the Naïve Bayes classifier was used as a reference, which performed practically identical to our classifier in all tests.

### Performance measures

We used a 10-fold cross-validation approach to evaluate the performance of the classifiers [32]. In brief, the dataset was randomly partitioned into 10 mutually exclusive subsets. For the validation, one of the 10 subsets was used as the test set and the other 9 subsets were combined to form a training set, and the whole process was repeated 10 times. In order to qualify and compare the performance of classifier, we calculated the receiver operating characteristic score, also know as area under ROC curve (AUC) [33]. All the test set predictions were combined and ordered by decreasing prediction score provided by classifier. Each instance in the combined test set was then assigned a binary class label by applying a threshold to the prediction score. The ROC curve was then generated by plotting the true positive rate against the false positive rate and the AUC was calculated by integrating the area under the ROC curve.

To asses if modification of the classifier results in improved performance, we compared the performance of two classifiers using paired t-test. The t-test assesses whether the means of two groups are statistically different from each other. Paired t-test was used as the same 10-fold partition of dataset was used for all classifiers. The calculation was carried out using t.test function from R [34].

## Authors' contributions

PW carried out the study and wrote the paper. AM contributed to the methodological design of the study and to drafting the paper. QZ contributed to the programming. AS conceived the study and participated in drafting the paper. BP conceived and designed the study, and participated in programming and writing the paper. All authors read and approved the final manuscript.

## Supplementary Material

Additional file 1 PubMed queries. PubMed queries to extract potentially IEDB relevant abstracts. Click here for file

Additional file 2 abstracts. The zip file contains four text files describing the IEDB relevance of PubMed abstracts used in this study (ac_id.txt: category A-C pathogen, allergen_id.txt: allergen, er_id.txt: emerging-reemerging diseases and other_id.txt: pathogens in other categories). Each line has three fields: PubMed PMID, relevance to IEDB and publication year. Click here for file
