# Supplementary material for: Automating document classification for the Immune Epitope Database
Source: BMC Bioinformatics. 2007 Jul 26;8:269. doi: 10.1186/1471-2105-8-269 (PMC1965490; doi:10.1186/1471-2105-8-269)
Supplement: Additional file 1 — PubMed queries. PubMed queries to extract potentially IEDB relevant abstracts. [file 1471-2105-8-269-S1.doc]

Mar 9, 2006

***Query for finding A-C Pathogen related abstracts that might contain epitope related data***

Changes made to the query

- added more epitope keywords like cd8, cd4, t cells, antibody reactivity, antigen repertoire
- added more pathogen specific keywords for Flu, yersinia, etc
- changed the publication date from Aug 2005 to end of Feb 2006
- removed the NOT keywords like cancer, autoimmune, hbv, hcv, etc.

**Query Parts**

1. Epitope Keywords
2. A-C Pathogen keywords
3. Filters
4. NOT keywords

(epitope[TW] OR epitopes[TW] OR mimotope[TW] OR ((MHC[tw] OR "major histocompatibility complex"[tw] OR HLA[tw]) AND (peptide[tw] OR peptides[tw])) OR "TCR recognition"[tw] OR ("Class"[tw] AND "I motif"[tw]) OR supermotif[tw] OR immunogenic linear OR ("peptide-based"[tw] AND CTL[tw]) OR phage displa*[tw] OR "antibody binding"[tw] OR "protective immune response"[tw] OR antibody recog*[tw] OR "cytotoxicity assay"[tw] OR "new monoclonal"[tw] OR "novel antibody"[tw] OR ( (monoclonal antibod*[tw]) AND "binding site"[tw]) OR ( (KA[tw] OR KD[tw]) AND (monoclonal[tw] OR mAb[tw])) OR "neutralizing antibody"[tw] OR "peptide vaccine"[tw] OR (peptide conjugate vaccine*[tw]) OR ((CD8[tw] OR CD4[tw]) AND “T cells”[tw] AND (peptide[tw] OR peptides[tw])) OR (“antigenic repertoire”[tw]) OR ((peptide[tw] OR peptides[tw]) AND “antibody reactivity”[tw]) OR ("Class II"[tw] AND (binding [tw] OR bound[tw] OR peptide[tw] OR peptides[tw])) OR "immunogenic peptide"[tw]) **AND** ((Anthrax[Text Word]) OR ("Bacillus anthracis"[Text Word]) OR ("B. anthracis"[Text Word]) OR (Botulism[Text Word]) OR ("Clostridium botulinum "[Text Word]) OR ("C. botulinum"[Text Word]) OR ("Brucella species"[Text Word]) OR ("Brucella sp"[Text Word]) OR (Brucellosis[Text Word]) OR ("Burkholderia mallei"[Text Word]) OR ("B. mallei"[Text Word]) OR (glanders[Text Word]) OR ("Burkholderia pseudomallei"[Text Word]) OR ("B. pseudomallei"[Text Word]) OR (melioidosis[Text Word]) OR (Cholera[Text Word]) OR ("Vibrio cholerae"[Text Word]) OR ("V. cholerae"[Text Word]) OR ("Clostridium perfringens"[Text Word]) OR ("C. perfringens"[Text Word]) OR ("Epsilon toxin"[Text Word]) OR ("Coxiella burnetii"[Text Word]) OR ("C. burnetii"[Text Word]) OR ("Q fever"[Text Word]) OR ("E. coli O157:H7"[Text Word]) OR ("Escherichia coli O157 H7"[Text Word]) OR ("Nipah virus"[Text Word]) OR (hantavirus[Text Word]) OR (hantaviruses[tw]) OR ("Francisella tularensis"[Text Word]) OR ("F. tularensis"[Text Word]) OR (tularemia[Text Word]) OR (Melioidosis[Text Word]) OR ("Burkholderia pseudomallei"[Text Word]) OR ("B. pseudomallei"[Text Word]) OR (Plague[Text Word]) OR ("Yersinia pestis"[Text Word]) OR ("Y. pestis"[Text Word]) OR (Psittacosis[Text Word]) OR (“Ricin”[Text Word] AND “toxin”[tw]) OR ("Ricinus communis"[Text Word]) OR ("R. communis"[Text Word]) OR ("Rickettsia prowazekii"[Text Word]) OR ("R. prowazekii"[Text Word]) OR ("typhus fever"[Text Word]) OR ("Salmonella species"[Text Word]) OR (“Salmonella sp”[Text Word]) OR (salmonellosis[Text Word]) OR ("Salmonella Typhi"[Text Word]) OR ("S. Typhi"[Text Word]) OR ("typhoid fever"[Text Word]) OR (Shigella[Text Word]) OR (shigellosis[Text Word]) OR (Smallpox[Text Word]) OR ("variola major"[Text Word]) OR ("Staphylococcal enterotoxin B"[Text Word]) OR (“Staphylococcal enterotoxin B”[tw]) OR ("Vibrio cholerae"[Text Word]) OR ("V. cholerae"[Text Word]) OR (cholera[Text Word]) OR (Alphavirus[Text Word]) OR ("Venezuelan equine encephalitis"[Text Word]) OR ("eastern equine encephalitis"[Text Word]) OR ("western equine encephalitis"[Text Word]) OR (Filovirus*[Text Word]) OR (Ebola[Text Word]) OR (Marburg[Text Word]) OR (Arenavirus[Text Word]) OR (Arenaviruses[Text Word]) OR (Lassa[Text Word]) OR (Machupo[Text Word]) OR (LCMV[Text Word]) OR (“Lymphocytic Choriomeningitis” [Text Word]) OR (junin[Text Word]) OR (guanarito[Text Word]) OR (RVF[Text Word]) OR (“Rift Valley Fever”[Text Word]) OR (bunyavirus[Text Word]) OR (Orthobunyavirus [Text Word]) OR (botulinum[Text Word]) OR (“Crimean-Congo” [Text Word]) OR (CCHF[Text Word]) OR (“Tick-borne encephalitis”[Text Word]) OR (“Tickborne encephalitis”[Text Word]) OR (TBE[Text Word]) OR (Rickettsiales [Text Word]) OR (rickettsias [Text Word]) OR (“alpha* proteobacteria”[Text Word]) OR (VEE[Text Word]) OR (WEE[Text Word]) OR (EEE[Text Word]) OR (“Diarrheagenic Escherichia coli”[Text Word]) OR (“Diarrheagenic E.coli”[Text Word]) OR (“pathogenic vibrio”[Text Word]) OR (“pathogenic vibrios”[Text Word]) OR (“Cyclospora cayetanensis”[Text Word]) OR (poxvirus*[Text Word]) OR (pox virus*[Text Word]) OR ("Cryptosporidium parvum"[Text Word]) OR ("C. parvum"[Text Word]) OR ("Influenza Virus"[Text Word]) OR ("Influenza A virus"[Text Word]) OR ("Influenza B virus"[Text Word]) OR ("Influenza C Virus"[Text Word]) OR ("Influenza D Virus"[Text Word]) OR ("Influenzavirus A"[Text Word]) OR ("Influenzavirus B"[Text Word]) OR ("Influenzavirus C"[Text Word]) OR ("dengue"[Text Word]) OR (influenza[Text Word]) OR (“yersinia enterocolitica”[Text Word]) OR (“y. enterocolitica”[Text Word]) OR (“flu”[Text Word]) OR (Flavivirus*[Text Word]) OR ("Dengue Hemorrhagic Fever"[Text Word]) OR ("dengue virus"[Text Word]) OR ("yellow fever"[Text Word]) OR ("YFV"[Text Word]) OR ("Tick-Borne"[Text Word]) OR ("Tuberculosis"[Text Word]) OR ("Rickettsia"[Text Word]) OR ("Rickettsia tsutsugamushi"[Text Word]) OR ("rhabdovirus"[Text Word]) OR ("rabies"[Text Word]) OR ("SARS"[Text Word]) OR ("SARS Coronavirus"[Text Word]) OR ("SARS Cov"[Text Word]) OR ("Listeria monocytogenes"[Text Word]) OR ("L. monocytogenes"[Text Word]) OR ("c. jejuni"[Text Word]) OR ("campylobacter jejuni"[Text Word]) OR (calicivirus*[Text Word]) OR ("hepatitis A"[Text Word]) OR ("hepatitis virus A"[Text Word]) OR ("giardia lamblia"[Text Word]) OR ("Giardiavirus"[Text Word]) OR ("entamoeba histolytica"[Text Word]) OR ("e. histolytica"[Text Word]) OR ("toxoplasma"[Text Word]) OR ("microsporidia"[Text Word]) OR ("west nile virus"[Text Word]) OR ("west nile"[Text Word]) OR ("LaCrosse Virus"[Text Word]) OR ("lac virus"[Text Word]) OR ("California encephalitis"[Text Word]) OR ("Japanese Encephalitis"[Text Word]) OR ("kyasanur forest disease"[Text Word]) OR ("KFDV"[Text Word])) **AND** (hasabstract[text] AND English[Lang] AND ("1900"[PDat]:"2006/02/28"[PDat])) **NOT** (Review[PT] OR Editorial[PT] OR meta-Analysis[PT] OR Comment[PT])

Mar 20, 06

**PubMed Query for Tuberculosis**

(epitope[TW] OR epitopes[TW] OR mimotope[TW] OR ((MHC[tw] OR "major histocompatibility complex"[tw] OR HLA[tw]) AND (peptide[tw] OR peptides[tw])) OR "TCR recognition"[tw] OR ("Class"[tw] AND "I motif"[tw]) OR supermotif[tw] OR immunogenic linear OR ("peptide-based"[tw] AND CTL[tw]) OR phage displa*[tw] OR "antibody binding"[tw] OR "protective immune response"[tw] OR antibody recog*[tw] OR "cytotoxicity assay"[tw] OR "new monoclonal"[tw] OR "novel antibody"[tw] OR ( (monoclonal antibod*[tw]) AND "binding site"[tw]) OR ( (KA[tw] OR KD[tw]) AND (monoclonal[tw] OR mAb[tw])) OR "neutralizing antibody"[tw] OR "peptide vaccine"[tw] OR (peptide conjugate vaccine*[tw]) OR ((CD8[tw] OR CD4[tw]) AND “T cells”[tw] AND (peptide[tw] OR peptides[tw])) OR (“antigenic repertoire”[tw]) OR ((peptide[tw] OR peptides[tw]) AND “antibody reactivity”[tw]) OR ("Class II"[tw] AND (binding [tw] OR bound[tw] OR peptide[tw] OR peptides[tw])) OR "immunogenic peptide"[tw]) **AND (**"Tuberculosis"[Text Word] OR "M. tuberculosis"[Text Word] OR "Bacillus tuberculosis"[Text Word] OR "Bacterium tuberculosis"[Text Word]**) AND (**hasabstract[text] AND English[Lang] AND ("1900"[PDAT] : "2005/12/31"[PDAT])**)** **NOT** **(**Review[PT] OR Editorial[PT] OR meta-Analysis[PT] OR Comment[PT]**)**

**PubMed Query for HIV**

(epitope[TW] OR epitopes[TW] OR mimotope[TW] OR ((MHC[tw] OR "major histocompatibility complex"[tw] OR HLA[tw]) AND (peptide[tw] OR peptides[tw])) OR "TCR recognition"[tw] OR ("Class"[tw] AND "I motif"[tw]) OR supermotif[tw] OR immunogenic linear OR ("peptide-based"[tw] AND CTL[tw]) OR phage displa*[tw] OR "antibody binding"[tw] OR "protective immune response"[tw] OR antibody recog*[tw] OR "cytotoxicity assay"[tw] OR "new monoclonal"[tw] OR "novel antibody"[tw] OR ( (monoclonal antibod*[tw]) AND "binding site"[tw]) OR ( (KA[tw] OR KD[tw]) AND (monoclonal[tw] OR mAb[tw])) OR "neutralizing antibody"[tw] OR "peptide vaccine"[tw] OR (peptide conjugate vaccine*[tw]) OR ((CD8[tw] OR CD4[tw]) AND “T cells”[tw] AND (peptide[tw] OR peptides[tw])) OR (“antigenic repertoire”[tw]) OR ((peptide[tw] OR peptides[tw]) AND “antibody reactivity”[tw]) OR ("Class II"[tw] AND (binding [tw] OR bound[tw] OR peptide[tw] OR peptides[tw])) OR "immunogenic peptide"[tw]) **AND (**"HIV"[Text Word] OR ("AIDS"[Text Word] AND "virus"[Text Word]) OR "Human immunodeficiency virus"[Text Word]**) AND (**hasabstract[text] AND English[Lang] AND ("1900"[PDAT] : "2005/12/31"[PDAT])**)** **NOT** **(**Review[PT] OR Editorial[PT] OR meta-Analysis[PT] OR Comment[PT]**)**

**PubMed Query for LCMV**

(epitope[TW] OR epitopes[TW] OR mimotope[TW] OR ((MHC[tw] OR "major histocompatibility complex"[tw] OR HLA[tw]) AND (peptide[tw] OR peptides[tw])) OR "TCR recognition"[tw] OR ("Class"[tw] AND "I motif"[tw]) OR supermotif[tw] OR immunogenic linear OR ("peptide-based"[tw] AND CTL[tw]) OR phage displa*[tw] OR "antibody binding"[tw] OR "protective immune response"[tw] OR antibody recog*[tw] OR "cytotoxicity assay"[tw] OR "new monoclonal"[tw] OR "novel antibody"[tw] OR ( (monoclonal antibod*[tw]) AND "binding site"[tw]) OR ( (KA[tw] OR KD[tw]) AND (monoclonal[tw] OR mAb[tw])) OR "neutralizing antibody"[tw] OR "peptide vaccine"[tw] OR (peptide conjugate vaccine*[tw]) OR ((CD8[tw] OR CD4[tw]) AND “T cells”[tw] AND (peptide[tw] OR peptides[tw])) OR (“antigenic repertoire”[tw]) OR ((peptide[tw] OR peptides[tw]) AND “antibody reactivity”[tw]) OR ("Class II"[tw] AND (binding [tw] OR bound[tw] OR peptide[tw] OR peptides[tw])) OR "immunogenic peptide"[tw]) **AND (**(LCMV[Text Word]) OR (“Lymphocytic Choriomeningitis” [Text Word])**) AND (**hasabstract[text] AND English[Lang] AND ("1900"[PDAT] : "2005/12/31"[PDAT])**)** **NOT** **(**Review[PT] OR Editorial[PT] OR meta-Analysis[PT] OR Comment[PT]**)**

**PubMed Query for Malaria (Plasmodium falciparum)**

(epitope[TW] OR epitopes[TW] OR mimotope[TW] OR ((MHC[tw] OR "major histocompatibility complex"[tw] OR HLA[tw]) AND (peptide[tw] OR peptides[tw])) OR "TCR recognition"[tw] OR ("Class"[tw] AND "I motif"[tw]) OR supermotif[tw] OR immunogenic linear OR ("peptide-based"[tw] AND CTL[tw]) OR phage displa*[tw] OR "antibody binding"[tw] OR "protective immune response"[tw] OR antibody recog*[tw] OR "cytotoxicity assay"[tw] OR "new monoclonal"[tw] OR "novel antibody"[tw] OR ( (monoclonal antibod*[tw]) AND "binding site"[tw]) OR ( (KA[tw] OR KD[tw]) AND (monoclonal[tw] OR mAb[tw])) OR "neutralizing antibody"[tw] OR "peptide vaccine"[tw] OR (peptide conjugate vaccine*[tw]) OR ((CD8[tw] OR CD4[tw]) AND “T cells”[tw] AND (peptide[tw] OR peptides[tw])) OR (“antigenic repertoire”[tw]) OR ((peptide[tw] OR peptides[tw]) AND “antibody reactivity”[tw]) OR ("Class II"[tw] AND (binding [tw] OR bound[tw] OR peptide[tw] OR peptides[tw])) OR "immunogenic peptide"[tw]) **AND (**("plasmodium falciparum"[Text Word]) OR ("P. falciparum"[Text Word]) OR ("malaria"[Text Word])**) AND (**hasabstract[text] AND English[Lang] AND ("1900"[PDAT] : "2005/12/31"[PDAT])**)** **NOT** **(**Review[PT] OR Editorial[PT] OR meta-Analysis[PT] OR Comment[PT]**)**

**Emerging and Re-emerging Pathogen Query (without SARS)**

**Query Parts**

1. Epitope Keywords
2. Emerging pathogen keywords
3. Filters
4. NOT keywords

(epitope[TW] OR epitopes[TW] OR mimotope[TW] OR ((MHC[tw] OR "major histocompatibility complex"[tw] OR HLA[tw]) AND (peptide[tw] OR peptides[tw])) OR "TCR recognition"[tw] OR ("Class"[tw] AND "I motif"[tw]) OR supermotif[tw] OR immunogenic linear OR ("peptide-based"[tw] AND CTL[tw]) OR phage displa*[tw] OR "antibody binding"[tw] OR "protective immune response"[tw] OR antibody recog*[tw] OR "cytotoxicity assay"[tw] OR "new monoclonal"[tw] OR "novel antibody"[tw] OR ( (monoclonal antibod*[tw]) AND "binding site"[tw]) OR ( (KA[tw] OR KD[tw]) AND (monoclonal[tw] OR mAb[tw])) OR "neutralizing antibody"[tw] OR "peptide vaccine"[tw] OR (peptide conjugate vaccine*[tw]) OR ((CD8[tw] OR CD4[tw]) AND “T cells”[tw] AND (peptide[tw] OR peptides[tw])) OR (“antigenic repertoire”[tw]) OR ((peptide[tw] OR peptides[tw]) AND “antibody reactivity”[tw]) OR ("Class II"[tw] AND (binding [tw] OR bound[tw] OR peptide[tw] OR peptides[tw])) OR "immunogenic peptide"[tw]) **AND** ((Acanthamebiasis[tw] or Acanthamoebae[tw]) or (“Australian bat lyssavirus”[tw] or ABL[tw]) or (“Babesia, atypical”[tw] or “atypical Babesia”[tw] or Babesiosis[tw]) or (“Bartonella henselae”[tw] or Bartonellosis[tw] or “Cat Scratch Fever”[tw]) or (Ehrlichiosis[tw] or Ehrlichia[tw]) or (“Encephalitozoon cuniculi”[tw] or “E. cuniculi”[tw]) or (“Encephalitozoon hellem”[tw] or “E. hellem”[tw]) or (“Enterocytozoon bieneusi”[tw] or “E. bieneusi”[tw]) or (“Helicobacter pylori”[tw] or “H. pylori”[tw]) or (“equine morbilli virus”[tw] or “Hendra virus”[tw] or “equine morbillivirus”[tw]) or (“Hepatitis C”[tw] or HCV[tw]) or (“Hepatitis E”[tw] or HEV[tw]) or (“Human herpesvirus 8”[tw] or HHV-8[tw] or KSHV[tw]) or (“Human herpesvirus 6”[tw] or HHV-6[tw]) or (“Lyme borreliosis”[tw] or “Borrelia burgdorferi”[tw] or “B. burgdorferi”[tw]) or (Microsporidia[tw] or Encephalitozoon[tw] or “Septata intestinalis”[tw] or Brachiola[tw] or Microsporidium[tw] or Nosema[tw] or Pleistophora[tw] or Trachipleistophora[tw] or Vittaforma[tw]) or (“Parvovirus B19”[tw] or ParvoB19[tw] or “Parvo B19”[tw]) or (“Enterovirus 71”[tw] or EV71[tw] or HEV71[tw]) or (“Prion disease”[tw] or “transmissible spongiform encephalopath*”[tw] or TSE[tw] or “bovine spongiform encephalopath*”[tw] or “Creutzfeldt-Jakob disease”[tw] or Scrapie[tw] or “chronic wasting disease”[tw]) or (“Streptococcus, group A”[tw] or “Streptococcus pyogenes”[tw] or “S. pyogenes”[tw] or “group A Streptococcus”[tw]) or (“Staphylococcus aureus”[tw] or “S. aureus”[tw]) or (“Coccidioides immitis”[tw] or Coccidioidomycosis[tw] or “C. immitis”[tw])) **AND** (hasabstract[text] AND English[Lang] AND ("1900"[PDat]:"2006/09/19"[PDat])) **NOT** (Review[PT] OR Editorial[PT] OR meta-Analysis[PT] OR Comment[PT])

**Allergy Pubmed search 7-20-2006**

(epitope[TW] OR epitopes[TW] OR mimotope[TW] OR ((MHC[tw] OR "major histocompatibility complex"[tw] OR HLA[tw]) AND (peptide[tw] OR peptides[tw])) OR "TCR recognition"[tw] OR ("Class"[tw] AND "I motif"[tw]) OR supermotif[tw] OR immunogenic linear OR ("peptide-based"[tw] AND CTL[tw]) OR phage displa*[tw] OR "antibody binding"[tw] OR "protective immune response"[tw] OR antibody recog*[tw] OR "cytotoxicity assay"[tw] OR "new monoclonal"[tw] OR "novel antibody"[tw] OR ( (monoclonal antibod*[tw]) AND "binding site"[tw]) OR ((KA[tw] OR KD[tw]) AND (monoclonal[tw] OR mAb[tw])) OR "neutralizing antibody"[tw] OR "peptide vaccine"[tw] OR (peptide conjugate vaccine*[tw]) OR ((CD8[tw] OR CD4[tw]) AND “T cell*”[tw] AND (peptide[tw] OR peptides[tw])) OR (“antigenic repertoire”[tw]) OR ((peptide[tw] OR peptides[tw]) AND “antibody reactivity”[tw]) OR ("Class II"[tw] AND (binding [tw] OR bound[tw] OR peptide[tw] OR peptides[tw])) OR "immunogenic peptide"[tw] or (monoclonal antibod*[tw] AND peptide*[tw]) or (T cell*[tw] AND peptide*[tw])) **AND** (allergy[tw] or allerg*[tw]or “[Immunoglobulin E](javascript:AL_get(this, 'subs', 'Immunoglobulin E');)”[tw] or IgE[tw] or “allergic rhinitis”[tw] or Hives[tw] or “Contact dermatitis”[tw] or “Atopic dermatitis”[tw] or eczema[tw] or Asthma[tw] or Conjunctivitis[tw] or Anaphyl*[tw] or “Immune Hypersensitivity”[tw] or “Chemical Sensitivity”[tw] or **“HAY FEVER”**[tw] **or Urticaria**[tw] or “Mast cell*”[tw] or Pollen[tw] or “Dust mite*”[tw] or Mold*[tw] or dander[tw] or [hypersensitivity](http://www.hon.ch/Library/Theme/Allergy/Glossary/hypersensitivity.html)[tw] or “[Celiac Disease](http://www.lib.uiowa.edu/hardin/md/celiacdisease.html)”[tw]or “Dermatitis Herpetiformis”[tw] or “[nettle rash](http://www.netdoctor.co.uk/diseases/facts/nettlerash.htm)”[tw] or “[allergic conjunctivitis](http://en.wikipedia.org/wiki/Allergic_conjunctivitis)”[tw] or atopic[tw] or Atopy[tw] or “seasonal [rhinitis](http://en.wikipedia.org/wiki/Rhinitis)”[tw] or “[contact dermatitis](http://www.intelihealth.com/IH/ihtIH/WSIHW000/7945/32104/351584.html?d=dmtContent#skin)”[tw] or “Serum sickness”[tw] or Allergens[MESH terms] or [Rhinitis, Allergic, Perennial](http://www.ncbi.nlm.nih.gov/entrez/query.fcgi?cmd=Retrieve&db=mesh&list_uids=68012221&dopt=Full)[MESH terms] or [Rhinitis, Allergic, Seasonal](http://www.ncbi.nlm.nih.gov/entrez/query.fcgi?cmd=Retrieve&db=mesh&list_uids=68006255&dopt=Full)[MESH terms] or [Allergens](javascript:AL_get(this, 'subs', 'Allergens');)[substance name]) **AND** (hasabstract[text] **AND** English[Lang] AND ("1900"[PDat]:"2006/07/20"[PDat])) **NOT** (Review[PT] OR Editorial[PT] OR meta-Analysis[PT] OR Comment[PT])

**Infectious Disease Query, for Hepatitis B**

**Add new epitope words**

**Query Parts**

1. Epitope Keywords
2. Infectious Disease keywords
3. Filters
4. NOT keywords

(epitope[TW] OR epitopes[TW] OR mimotope[TW] OR ((MHC[tw] OR major histocompatibility complex[tw] OR HLA[tw]) AND (peptide[tw] OR peptides[tw])) OR TCR recognition[tw] OR (Class[tw] AND I motif[tw]) OR supermotif[tw] OR (peptide-based[tw] AND CTL[tw]) OR phage displa*[tw] OR antibody binding[tw] OR protective immune response[tw] OR antibody recog*[tw] OR cytotoxicity assay[tw] OR new monoclonal[tw] OR novel antibody[tw] OR ((monoclonal antibod*[tw]) AND binding site[tw]) OR ((KA[tw] OR KD[tw]) AND (monoclonal[tw] OR mAb[tw])) OR neutralizing antibod*[tw] OR peptide vaccine[tw] OR peptide conjugate vaccin*[tw] OR ((CD8[tw] OR CD4[tw]) AND T cell*[tw] AND (peptide[tw] OR peptides[tw])) OR antigenic repertoire[tw] OR ((peptide[tw] OR peptides[tw]) AND antibody reactivity[tw]) OR (Class II[tw] AND (binding [tw] OR bound[tw] OR peptide[tw] OR peptides[tw])) OR "immunogenic peptide"[tw] or (monoclonal antibod*[tw] and peptide*[tw]) or (T cell*[tw] and peptide*[tw])) AND (“Hepatitis B”[tw] or "Hepatitis B virus"[MeSH Terms]) AND hasabstract[text] AND English[Lang] AND ("1900"[PDat]:"2007/01/18"[PDat]) **NOT** (Review[PT] OR Editorial[PT] OR meta-Analysis[PT] OR Comment[PT] or pubstatusaheadofprint[ALL])
